# Supplementary material for: The Effect of Lameness on Milk Production of Dairy Goats
Source: Animals (Basel). 2023 May 23;13(11):1728. doi: 10.3390/ani13111728 (PMC10251923; doi:10.3390/ani13111728)
Supplement: Supplementary file 1 [file animals-13-01728-s001.zip › Table S1.pdf]

Supplementary Table

Table S1. Locomotion scoring strategy used to measure lameness in dairy goats (adapted from Deeming et al. 2018 [26]).

Severity Score	Label	Clinically Lame	Limping	Gait Description	Modifications
0	Normal/not lame	No	No	Moving forward with even strides where hooves track up. Weight-bearing and no apparent head nodding.	
1	Uneven gait	No	Uneven	Moving forward with shorter strides where hooves do not track up. Weight-bearing, and having an absent head nodding, however, have joints that may show stiffness.	
2	Mildly lame	No	Yes	Moving forward with shorter strides where hooves do not track up. One or more legs/feet may be affected. Weight-bearing, and having an absent head nodding, however, may show a mild limp.	
3	Moderately lame	Yes	Yes	Reluctant to move forward and may display a moderate limp. One or more legs could be affected and may display some goose-stepping.	
4	Severely lame	Yes	Yes	Refusal to bear any weight on one foot. Severe limping or extreme goose-stepping or walking on the knees.	If there is no limp, but are very reluctant to move forward and weight-bear due to multiple feet involved.
